# INTERACTIONS WITH PEOPLE WITH DEMENTIA, LEARNING EXPERIENCES, AND PUBLIC STIGMA AGAINST DEMENTIA

**DOI:** 10.1093/geroni/igac059.2006

**Published:** 2022-12-20

**Authors:** Taiji Noguchi, Takeshi Nakagawa, Ayane Komatsu, Erhua Shang, Chiyoe Murata, Tami Saito

**Affiliations:** National Center for Geriatrics and Gerontology, Obu, Aichi, Japan; National Center for Geriatrics and Gerontology, Obu, Aichi, Japan; National Center for Geriatrics and Gerontology, Obu, Aichi, Japan; Aichi Toho University, Nagoya, Aichi, Japan; Tokai Gakuen University, Nagoya, Aichi, Japan; National Center for Geriatrics and Gerontology, Obu, Aichi, Japan

## Abstract

Overcoming stigma around dementia is a global challenge. This cross-sectional study examined the association of experiences of interacting with people with dementia (PwD) and learning about dementia, with the public-stigma against dementia. We recruited—via an internet survey—710 Japanese adults (mean age = 46.3 years; 49.3% females) without any medical or welfare license, or dementia-related work experience. Public-stigma against dementia was assessed using the Japanese version of Phillipson et al.’s scale (2012) exploring dementia-related attitudes in the context of “personal avoidance,” “person centeredness,” “fear of labeling,” and “fear of discrimination.” Multivariable linear regression analysis was employed to examine the association of interacting with PwD and learning regarding dementia as explanatory variables with dementia stigma score, adjusting for sociodemographic variables. Regarding interactions with PwD, talking or activities with PwD were associated with low “personal avoidance” (β = -1.47, p = 0.002), “fear of labeling” (β = -0.96, p = 0.020), and “fear of discrimination” (β = -0.396, p = 0.043). Experiences of living with PwD were associated with low “personal avoidance” (β = -2.35, p = 0.002) and “fear of discrimination” (β = -0.789, p = 0.013). Learning experiences at school regarding dementia were associated with low “personal avoidance” (β = -4.01, p < 0.001), and self-learning experiences were associated with low “personal avoidance” (β = -1.73, p = 0.049) and high “person centeredness” (β = 1.27, p = 0.037). However, workplace learning was not associated with any area. Interacting with PwD and learning about dementia might reduce associated public-stigma.

